# μ_3_-Chlorido-tris­(bis­{1-[2-(dimethyl­amino)­eth­yl]-3-methyl­imidazol-2-yl­idene}silver(I)) dichloride

**DOI:** 10.1107/S1600536812004473

**Published:** 2012-02-10

**Authors:** Christoph Topf, Sebastian Leitner, Uwe Monkowius

**Affiliations:** a Johannes Kepler Universität Linz, Institut für Anorganische Chemie, Altenbergerstrasse 69, A-4040 Linz, Austria

## Abstract

In the crystal structure of the title compound, [Ag_3_Cl(C_8_H_15_N_3_)_6_]Cl_2_, the Ag^I^ ion, which is located on a twofold rotation axis, exists in a T-shape coordination environment. Two carbene C atoms of the N-heterocyclic carbene (NHC) ligands are bonded tightly forming a slightly bent [Ag(NHC)_2_]^+^ cation [C—Ag—C angle = 162.80 (18)°]. Three of these complex cations are further aggregated by one bridging chloride anion, which is lying on a threefold rotoinversion axis and is only loosely binding to the Ag^+^ ions. The N atom of the amine group is not engaged in any coordinative bond.

## Related literature

For related literature concerning similar N-heterocyclic carbenes, see: Topf, Hirtenlehner, Fleck *et al.* (2011 ▶); Topf, Hirtenlehner & Monkowius (2011 ▶); Leitner *et al.* (2011 ▶). For related structures, see: Hirtenlehner *et al.* (2011 ▶); Wang *et al.* (2006 ▶). For details of the preparation, see: Topf, Hirtenlehner, Zabel *et al.* (2011 ▶).
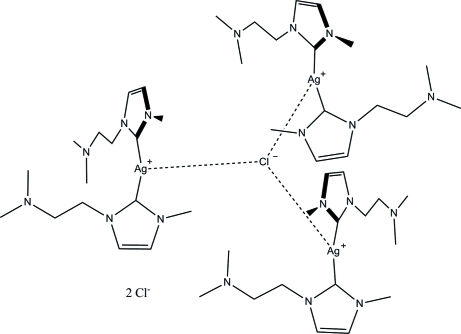


## Experimental

### 

#### Crystal data

[Ag_3_Cl(C_8_H_15_N_3_)_6_]Cl_2_
*M* *_r_* = 1349.34
Trigonal, 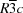

*a* = 12.7300 (16) Å
*c* = 66.789 (12) Å
*V* = 9373 (2) Å^3^
*Z* = 6
Mo *K*α radiation
μ = 1.11 mm^−1^
*T* = 200 K
0.50 × 0.36 × 0.31 mm

#### Data collection

Bruker SMART X2S diffractometer
Absorption correction: multi-scan (*SADABS*; Bruker, 2009 ▶) *T*_min_ = 0.61, *T*_max_ = 0.73
18593 measured reflections
1859 independent reflections
1590 reflections with *I* > 2σ(*I*)
*R*_int_ = 0.060

#### Refinement

*R*[*F* ^2^ > 2σ(*F* ^2^)] = 0.034
*wR*(*F* ^2^) = 0.095
*S* = 1.03
1859 reflections
113 parameters
H-atom parameters constrained
Δρ_max_ = 1.28 e Å^−3^
Δρ_min_ = −0.46 e Å^−3^

### 

Data collection: *APEX2* and *GIS* (Bruker, 2009 ▶); cell refinement: *SAINT* (Bruker, 2009 ▶); data reduction: *SAINT*; program(s) used to solve structure: *SHELXS97* (Sheldrick, 2008 ▶); program(s) used to refine structure: *SHELXL97* (Sheldrick, 2008 ▶); molecular graphics: *ORTEP-3* (Farrugia, 1997 ▶); software used to prepare material for publication: *publCIF* (Westrip, 2010 ▶).

## Supplementary Material

Crystal structure: contains datablock(s) global, I. DOI: 10.1107/S1600536812004473/bt5811sup1.cif

Structure factors: contains datablock(s) I. DOI: 10.1107/S1600536812004473/bt5811Isup2.hkl

Supplementary material file. DOI: 10.1107/S1600536812004473/bt5811Isup3.cdx

Additional supplementary materials:  crystallographic information; 3D view; checkCIF report

## Figures and Tables

**Fig. 1.**
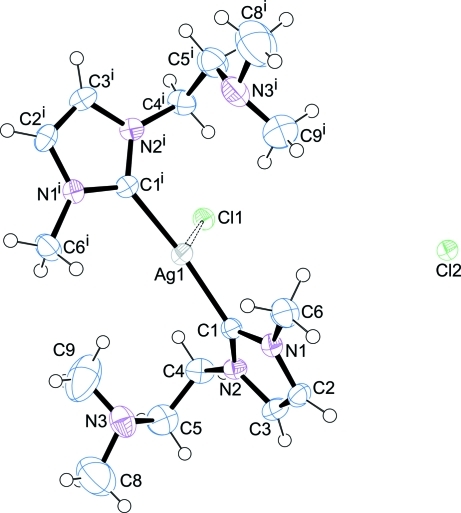
View of the title compound with the atom numbering scheme (symmetry code: (i) x-y + 1/3, -y + 2/3, -z + 1/6). Displacement ellipsoids for non-H atoms are drawn at the 50% probability level.

**Fig. 2.**
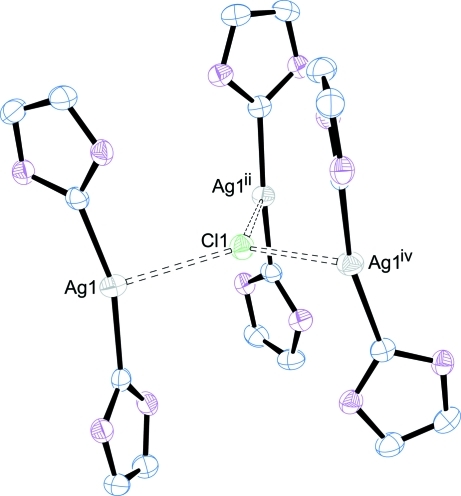
[Ag3Cl]2+ cation in the crystals of the title compound. The H atoms and the methyl and 2-dimethyl-amino-ethyl groups are omitted for the sake of clarity (symmetry codes: (ii) -y + 1, x-y, z; (iv) y + 1/3, x - 1/3, -z + 1/6).

## supplementary crystallographic information

### Comment

In the course of our studies on gold- and silver-complexes bearing functionalized N-heterocyclic carbenes (NHCs), we became interested in examples with amino groups containing side arms at a nitrogen atom of the NHC ligands (Topf, Hirtenlehner, Fleck *et al.* (2011); Topf, Hirtenlehner & Monkowius (2011); Leitner *et al.*, 2011; Hirtenlehner *et al.*, 2011). Just recently, we published the multifarious coordination patterns of such silver complexes (Topf, Hirtenlehner, Zabel *et al.*, 2011): E.g., in the ionic compound [(C_8_H_15_N_3_)_2_Ag][AgCl_2_], which is formed from the respective imidazolium chloride and Ag_2_O in dichloromethane, the ions are aggregated to infinite chains with short silver-silver contacts. Treatment of this complex with HBF_4_ yields the cluster (C_8_H_15_N_3_)_4_Ag_10_Cl_10_ with the carbene carbon atom binding in a unusual µ_2_-fashion to two silver atoms. In an attempt to prepare this cluster, crystals of the title compound were formed representing the third silver chloride complex in the series of this ligand. The formation of this complex is easily rationalized by the precipitation of AgCl from [(C_8_H_15_N_3_)_2_Ag][AgCl_2_] in solution.

The silver atom is in a slightly bent linear coordination with an Ag1—C1 bond length of 2.099 (3) Å and an angle C1—Ag1—C1^i^ of 162.8 (2)°. Perpendicular to the C1—Ag1—C1^i^ vector, a chloride anion is loosely binding with an Ag1—Cl1 bond length of 2.981 (1) Å. The chloride Cl1 is linking three [(C_8_H_15_N_3_)_2_Ag]^+^ units in a µ_3_-fashion forming a *D*_3_ symmteric trimeric aggregate. The net 2+ charge is balanced by two non-interacting chloride ions. Within other cationic species of the type [(NHC)_2_Ag]^+^, the imidazole ring planes are usually found in a coplanar arrangement due to a higher π-backbonding contribution compared to a perpendicular orientation. Presumably because of steric reasons, the [(C_8_H_15_N_3_)_2_Ag]^+^ moiety features an arrangement with both imidazole ring planes approaching a perpendicular orientation [N1—C1—C1^i^—N1^i^ 89.8°]. The distance between two silver atoms within the trimer is 5.164 Å, which is well beyond the range of argentophilic interactions. It should be noted, that this aggregation pattern is very rare and to the best of our knowledge reported only for {[(NHC)_2_Ag]_3_(µ_3_-I)}I_2_ (NHC = 1-methyl-3-picolyl-imidazol-2-ylidene) (Wang *et al.*, 2006) and {[(NHC)_2_Au]_3_(µ_3_-Br)}Br_2_ (NHC= 1-methyl-3-benzyl-imidazol-2-ylidene) (Hirtenlehner *et al.*, 2011).

### Experimental

Crystals of the title compound were formed in an attempt to synthesize the silver cluster (C_8_H_15_N_3_)_4_Ag_10_Cl_10_ according to a literature procedure (Topf, Hirtenlehner, Zabel *et al.*, 2011).

### Refinement

The hydrogen atoms were placed in calculated positions with C—H = 0.95–0.99 Å and refined using a riding model with *U*_iso_(H) = 1.5 *U*_eq_(C) for methyl groups and *U*_iso_(H) = 1.2 *U*_eq_(C) for methylen and aromatic hydrogen atoms. The highest residual electron density peak is located 1.28 Å from H9A and the deepest hole is located 0.53 Å from C9.

### Crystal data

**Table:**

[Ag_3_Cl(C_8_H_15_N_3_)_2_]Cl_2_	*F*(000) = 4176
*M**_r_* = 1349.34	*D*_x_ = 1.434 Mg m^−^^3^
Trigonal, *R*3*c*	Mo *K*α radiation, λ = 0.71073 Å
*a* = 12.7300 (16) Å	µ = 1.11 mm^−^^1^
*c* = 66.789 (12) Å	*T* = 200 K
*V* = 9373 (2) Å^3^	Prism, colourless
*Z* = 6	0.50 × 0.36 × 0.31 mm

### Data collection

**Table:**

Bruker SMART X2S diffractometer	1859 independent reflections
Radiation source: sealed MicroFocus tube	1590 reflections with *I* > 2σ(*I*)
Doubly curved silicon crystal monochromator	*R*_int_ = 0.060
ω scans	θ_max_ = 25.1°, θ_min_ = 3.1°
Absorption correction: multi-scan (*SADABS*; Bruker, 2009)	*h* = −15→14
*T*_min_ = 0.61, *T*_max_ = 0.73	*k* = −15→15
18593 measured reflections	*l* = −79→79

### Refinement

**Table:**

Refinement on *F*^2^	Primary atom site location: structure-invariant direct methods
Least-squares matrix: full	Secondary atom site location: difference Fourier map
*R*[*F*^2^ > 2σ(*F*^2^)] = 0.034	Hydrogen site location: inferred from neighbouring sites
*wR*(*F*^2^) = 0.095	H-atom parameters constrained
*S* = 1.03	*w* = 1/[σ^2^(*F*_o_^2^) + (0.0504*P*)^2^ + 47.4314*P*] where *P* = (*F*_o_^2^ + 2*F*_c_^2^)/3
1859 reflections	(Δ/σ)_max_ = 0.001
113 parameters	Δρ_max_ = 1.28 e Å^−^^3^
0 restraints	Δρ_min_ = −0.46 e Å^−^^3^

### Special details

**Table:**

Geometry. All e.s.d.'s (except the e.s.d. in the dihedral angle between two l.s. planes) are estimated using the full covariance matrix. The cell e.s.d.'s are taken into account individually in the estimation of e.s.d.'s in distances, angles and torsion angles; correlations between e.s.d.'s in cell parameters are only used when they are defined by crystal symmetry. An approximate (isotropic) treatment of cell e.s.d.'s is used for estimating e.s.d.'s involving l.s. planes.
Refinement. Refinement of *F*^2^ against ALL reflections. The weighted *R*-factor *wR* and goodness of fit *S* are based on *F*^2^, conventional *R*-factors *R* are based on *F*, with *F* set to zero for negative *F*^2^. The threshold expression of *F*^2^ > σ(*F*^2^) is used only for calculating *R*-factors(gt) *etc*. and is not relevant to the choice of reflections for refinement. *R*-factors based on *F*^2^ are statistically about twice as large as those based on *F*, and *R*- factors based on ALL data will be even larger.

### Fractional atomic coordinates and isotropic or equivalent isotropic displacement parameters (Å^2^)

**Table:**

	*x*	*y*	*z*	*U*_iso_*/*U*_eq_	
Ag1	0.90085 (3)	0.3333	0.0833	0.03038 (16)	
C6	1.1504 (3)	0.5164 (4)	0.05466 (6)	0.0422 (9)	
H6A	1.1374	0.5764	0.0617	0.063*	
H6B	1.2184	0.558	0.0453	0.063*	
H6C	1.1689	0.4703	0.0644	0.063*	
C1	0.9354 (3)	0.3530 (3)	0.05243 (5)	0.0257 (7)	
N2	0.8610 (3)	0.2965 (3)	0.03679 (4)	0.0284 (6)	
C5	0.7252 (4)	0.0798 (4)	0.03044 (7)	0.0543 (11)	
H5A	0.6394	0.0146	0.0315	0.065*	
H5B	0.7471	0.0909	0.0161	0.065*	
N1	1.0411 (2)	0.4340 (2)	0.04359 (4)	0.0271 (6)	
C4	0.7366 (3)	0.1961 (4)	0.03843 (6)	0.0388 (9)	
H4A	0.682	0.2158	0.0308	0.047*	
H4B	0.7112	0.1848	0.0526	0.047*	
C3	0.9192 (3)	0.3417 (3)	0.01870 (6)	0.0358 (9)	
H3	0.8853	0.3163	0.0057	0.043*	
C2	1.0327 (3)	0.4284 (3)	0.02306 (5)	0.0341 (8)	
H2	1.095	0.4766	0.0138	0.041*	
N3	0.8016 (3)	0.0410 (3)	0.04089 (6)	0.0475 (9)	
C8	0.7953 (7)	−0.0580 (6)	0.02927 (11)	0.101 (2)	
H8A	0.842	−0.0898	0.036	0.151*	
H8B	0.829	−0.0286	0.0159	0.151*	
H8C	0.7104	−0.1226	0.028	0.151*	
C9	0.7645 (7)	0.0065 (6)	0.06074 (11)	0.114 (3)	
H9A	0.772	0.0761	0.0682	0.172*	
H9B	0.8157	−0.0217	0.067	0.172*	
H9C	0.6797	−0.0591	0.0609	0.172*	
Cl1	0.6667	0.3333	0.0833	0.0307 (4)	
Cl2	0.3333	0.6667	0.00857 (2)	0.0344 (3)	

### Atomic displacement parameters (Å^2^)

**Table:**

	*U*^11^	*U*^22^	*U*^33^	*U*^12^	*U*^13^	*U*^23^
Ag1	0.0353 (2)	0.0290 (2)	0.0247 (2)	0.01451 (11)	0.00056 (7)	0.00112 (14)
C6	0.032 (2)	0.035 (2)	0.048 (2)	0.0078 (17)	−0.0022 (17)	−0.0035 (17)
C1	0.0288 (17)	0.0288 (17)	0.0267 (18)	0.0197 (15)	−0.0005 (14)	0.0007 (13)
N2	0.0256 (14)	0.0345 (16)	0.0289 (16)	0.0180 (13)	0.0007 (12)	−0.0018 (12)
C5	0.040 (2)	0.053 (3)	0.057 (3)	0.013 (2)	−0.003 (2)	−0.005 (2)
N1	0.0246 (14)	0.0259 (14)	0.0314 (15)	0.0131 (12)	0.0013 (11)	0.0016 (12)
C4	0.0238 (18)	0.046 (2)	0.044 (2)	0.0161 (17)	−0.0016 (15)	−0.0062 (18)
C3	0.040 (2)	0.048 (2)	0.0245 (19)	0.0255 (18)	0.0000 (15)	0.0000 (15)
C2	0.039 (2)	0.039 (2)	0.0290 (19)	0.0233 (17)	0.0095 (15)	0.0072 (15)
N3	0.0411 (19)	0.0327 (18)	0.061 (2)	0.0126 (15)	−0.0096 (17)	−0.0030 (16)
C8	0.108 (5)	0.066 (4)	0.121 (6)	0.039 (4)	0.011 (4)	−0.010 (4)
C9	0.132 (7)	0.081 (5)	0.069 (4)	0.007 (4)	−0.034 (4)	0.012 (3)
Cl1	0.0299 (6)	0.0299 (6)	0.0323 (10)	0.0150 (3)	0	0
Cl2	0.0356 (5)	0.0356 (5)	0.0319 (7)	0.0178 (3)	0	0

### Geometric parameters (Å, º)

**Table:**

Ag1—C1^i^	2.099 (3)	N1—C2	1.374 (5)
Ag1—C1	2.099 (3)	C4—H4A	0.99
C6—N1	1.458 (5)	C4—H4B	0.99
C6—H6A	0.98	C3—C2	1.340 (5)
C6—H6B	0.98	C3—H3	0.95
C6—H6C	0.98	C2—H2	0.95
C1—N2	1.350 (5)	N3—C9	1.402 (8)
C1—N1	1.355 (4)	N3—C8	1.447 (7)
N2—C3	1.383 (5)	C8—H8A	0.98
N2—C4	1.459 (5)	C8—H8B	0.98
C5—N3	1.469 (6)	C8—H8C	0.98
C5—C4	1.511 (6)	C9—H9A	0.98
C5—H5A	0.99	C9—H9B	0.98
C5—H5B	0.99	C9—H9C	0.98
			
C1^i^—Ag1—C1	162.80 (18)	N2—C4—H4B	109.4
N1—C6—H6A	109.5	C5—C4—H4B	109.4
N1—C6—H6B	109.5	H4A—C4—H4B	108.0
H6A—C6—H6B	109.5	C2—C3—N2	106.6 (3)
N1—C6—H6C	109.5	C2—C3—H3	126.7
H6A—C6—H6C	109.5	N2—C3—H3	126.7
H6B—C6—H6C	109.5	C3—C2—N1	106.5 (3)
N2—C1—N1	103.5 (3)	C3—C2—H2	126.8
N2—C1—Ag1	130.4 (3)	N1—C2—H2	126.8
N1—C1—Ag1	126.0 (2)	C9—N3—C8	111.8 (5)
C1—N2—C3	111.5 (3)	C9—N3—C5	112.2 (5)
C1—N2—C4	125.0 (3)	C8—N3—C5	106.3 (4)
C3—N2—C4	123.4 (3)	N3—C8—H8A	109.5
N3—C5—C4	114.0 (3)	N3—C8—H8B	109.5
N3—C5—H5A	108.8	H8A—C8—H8B	109.5
C4—C5—H5A	108.8	N3—C8—H8C	109.5
N3—C5—H5B	108.8	H8A—C8—H8C	109.5
C4—C5—H5B	108.8	H8B—C8—H8C	109.5
H5A—C5—H5B	107.7	N3—C9—H9A	109.5
C1—N1—C2	111.9 (3)	N3—C9—H9B	109.5
C1—N1—C6	123.7 (3)	H9A—C9—H9B	109.5
C2—N1—C6	124.4 (3)	N3—C9—H9C	109.5
N2—C4—C5	111.3 (3)	H9A—C9—H9C	109.5
N2—C4—H4A	109.4	H9B—C9—H9C	109.5
C5—C4—H4A	109.4		

Symmetry code: (i) *x*−*y*+1/3, −*y*+2/3, −*z*+1/6.
